# Individual Differences in the Effects of Life Events on Personality Trait Change

**DOI:** 10.1111/jopy.70055

**Published:** 2026-02-26

**Authors:** Lara Oeltjen, Christian Kandler

**Affiliations:** ^1^ Department of Psychology University of Bremen Bremen Germany; ^2^ Department of Psychology Bielefeld University Bielefeld Germany

**Keywords:** individual differences, life event perceptions, life events, personality trait change

## Abstract

**Objective:**

To date, effects of life events on personality trait change have been unsystematic, tenuous, and difficult to replicate. We focus on individual differences in change processes following life events, which have been neglected in previous studies.

**Method:**

Based on a novel Moderated Nonlinear Latent‐State–Trait modeling approach we investigate the impact of the occurrence and individual perception of life events on different types of self‐ and informant‐reported personality trait change, considering various moderators. A total of 623 participants with up to three informants per target provided data across three assessment waves at 2‐year intervals.

**Results:**

Several event effects on mean‐level personality change and change in trait variability emerged; however, these depended on age, gender, the individual perception of life events, the type of trait change, the time‐lag between event occurrence and personality assessment, the repeated occurrence of life events, and the personality assessment method.

**Conclusions:**

This high volatility of life‐event effects might serve as an explanation for the inconclusive state of research on this topic, as studies differing in one or more of these factors are unlikely to produce similar results.

1

Personality traits, commonly defined as characteristic patterns of thinking, feeling, striving, and acting that distinguish individuals from each other (Baumert et al. 2017) are associated with a great number of real‐life outcomes, including job performance (e.g., Dudley et al. 2006) and physical and mental health (e.g., Hampson et al. 2007; Hopwood et al. 2012). Today, it is widely acknowledged that personality traits (although defined as relatively stable entities) can also change over the entire lifespan (Wundrack et al. 2021). Personality changes can in turn lead to changes in real‐life outcomes (Bleidorn et al. 2020), so understanding their causes and processes is of great importance for many research areas beyond personality psychology.

From early on, major life events (LE) were discussed as one important predictor of personality development (Bühler, Orth, et al. 2024). LE refers to time‐discrete transitions in one's life that mark the beginning or end of a certain status quo, are experienced as personally significant and are often accompanied by changes in social roles (Luhmann et al. 2012, 2021; Specht 2017), which makes them plausible candidates for drivers of personality change. However, empirical results to date have been inconclusive, with effects being rather small and inconsistent across studies, which is often presumed to be the consequence of inadequate research designs and analyses: Many former studies do not use a prospective design or rely on simple pre–post comparisons without a nonevent control group, neglect potentially confounding variables such as age and rely exclusively on self‐reports of personality (Bleidorn et al. 2018; Bühler, Orth, et al. 2024; Specht et al. 2011). There is also a strong focus on mean‐level changes while other types of change and interindividual differences in change processes are neglected (Jackson and Beck 2021). Potentially important moderators of responses to LE, such as their individual perception, that could explain interindividual differences, are understudied (Rakhshani et al. 2021).

With this study, we aim to overcome several limitations of previous research to investigate the effects of LE on trait change more comprehensively. First, we apply a novel (latent) modeling approach of personality change, Moderated Nonlinear Latent State Trait Modeling (MNLST, Oeltjen et al. 2023), which allows us to investigate different types of personality change following LE in one comprehensive model while controlling for potentially confounding covariates. Second, we use self‐ and informant reports as indicators of personality and compare both perspectives with respect to trait change in response to LE. Third, we incorporate important moderators of the effects of LE on personality change, most importantly their individual perception. Fourth, we examine effects of LE over different time intervals and also consider the repeated exposure to LE. Fifth, we use a novel assessment approach of broader but homogenous LE categories that allows us to incorporate a wide range of events from five life domains without requiring the events to be aggregated.

In the following, we first review the theoretical perspectives and empirical evidence on the role of LE in personality trait change. Based on the limitations of former studies, we outline our own research design that aims to extend previous research on this topic in several ways. We investigate our research questions with data from a multirater longitudinal study covering three measurement occasions over a 4‐year period. Finally, we discuss the implications of our results and possible limitations of our study and derive recommendations for future research.

### 1.1 Sources of Personality Change

Although traits were long considered as fundamentally stable entities (Kandler and Rauthmann 2022), it is widely acknowledged today that they can actually change over the entire lifespan (Wundrack et al. 2021) and the search for drivers of these change processes has recently began (Wagner et al. 2020). There are several theories about how and why personality traits change. The Five Factor theory of personality (McCrae and Costa Jr. 2008), for instance, considers trait change to be completely explained by biological maturation processes. And indeed, there is evidence of universal developmental trends consistent across cultures towards greater maturity, meaning that up to the age of 30 neuroticism decreases while Agreeableness and Conscientiousness increase (Atherton et al. 2020). In old age, however, this trend is reversed (Atherton et al. 2020), and empirical evidence also indicates substantial differences between cultures in the timing of these changes, associated with the culture‐specific timing of major life transitions (Bleidorn et al. 2013). Moreover, individual trait differences are of limited stability over time suggesting that change trajectories differ between people (Bühler, Orth, et al. 2024). As not all people go through the same life transitions (e.g., get a job, enter a relationship or become a parent) at the same time or at all, not all people change in line with overall age trends (Lüdtke et al. 2011). Those findings question the theory of universal personality change patterns fully determined by a genetic maturation program and strengthen the role that individual environmental factors such as LE may play in personality change.

### 1.2 Personality Change Following Life Events

From a theoretical perspective, it is very plausible that LE have the potential to affect personality trait change, as they are often accompanied by changes in social networks (e.g., due to a move, entering university, or starting a new job) and investments in new social roles (e.g., becoming an employee, a romantic partner or a parent). These social structures convey new behavioral expectations including cognitive and emotional demands and offer corresponding feedback in the form of reward and punishment structures (Atherton et al. 2020). In trying to fulfill these demands, people can modify their personality states. If states are repeated, they may become habits, generalize across situations and finally (in a bottom‐up fashion) result in enduring personality changes (Roberts et al. 2005; Roberts and Jackson 2008; Wrzus and Roberts 2017).

Despite the theoretical plausibility of LE as drivers of personality change, the current state of empirical evidence remains inconclusive. In recent years, four systematic reviews and meta‐analyses (Bleidorn et al. 2018; Bühler, Orth, et al. 2024; Haehner et al. 2025; Specht 2017) have been conducted to identify patterns and consistencies in the effects of LE on personality trait change. A summary of the effects identified in these reviews can be found in Table 1. The Table shows that although some effects could be found in each of these studies, there is only little overlap in the identified effects. No effect was consistently found across all four reviews. Only five effects (printed in bold type) emerged in at least three of the four reviews: the effects of work life entry on Conscientiousness (positive) and Neuroticism (negative), the positive effects of divorce on Openness and Agreeableness, and the positive effect of starting a new relationship on Extraversion. Most of the identified effects vary across studies. Specht (2017) and Bleidorn et al. (2018) even reported opposing effects of starting a new relationship on Agreeableness. The most recent coordinated data (meta‐)analysis of Haehner et al. (2025) across seven large‐scale panel studies (*N* = 196,256) found only small effects.

**TABLE 1:** Overview of the results of systematic reviews and meta‐analyses.

Life events	Openness				Conscientiousness				Extraversion				Agreeableness				Neuroticism			
	Spe	Ble	Büh	Hae	Spe	Ble	Büh	Hae	Spe	Ble	Büh	Hae	Spe	Ble	Büh	Hae	Spe	Ble	Büh	Hae
Work																				
Graduation		+			+	+			−					+			−	−		
Work life entry					**+**	**+**		**+**			+					+	**−**		**−**	**−**
Job change	~								~											
Promotion	+				+												−			
Unemployment	−				−								−							
Retirement					−															
Love																				
New relationship							+		**+**	**+**	**+**		−	+			−		−	
Marriage	−			−					−				−				−			
Childbirth				−	−			−	−											
Seperation	+			+									+			+				−
Divorce	**+**		**+**	**+**									**+**		**+**	**+**			+	
Widowhood												−								
Health	−				−				−								+			

*Note:* Spe: Results of the review of Specht (2017), Ble: Results of review of Bleidorn et al. (2018), Büh: Results of the review of Bühler, Orth, et al. (2024), Hae: Postevent results of the meta‐analysis of Haehner et al. (2025). +: increase after the respective LE, −: decrease after the respective LE, ~: direction of effect depends on moderator, white space: no robust effect, gray space: LE not included in review. Effects found in at least three of the four reviews/meta‐analyses are printed in bold type.

### 1.3 Limitations of Previous Research

Beyond the authors of the studies mentioned above, many personality researchers have noted that findings in this research area are mixed, replicable effects are sparse, and the current state of evidence is inconclusive (e.g., Haehner et al. 2022; Jackson and Beck 2021; Rakhshani et al. 2021; Wundrack et al. 2021). However, this does not seem surprising, as previous studies often used limited research designs and were often not originally designed to investigate the effects of LE on personality trait change (Bleidorn et al. 2018).

The lack of prospective studies with more than two measurement occasions (Bleidorn et al. 2018) hinders the investigation of nonlinear change trajectories and time‐dependent effects, which have already been found for LE (Luhmann et al. 2012). Moreover, many studies do not involve a nonevent comparison group (two thirds in the recent meta‐analysis of Bühler, Orth, et al. 2024) and do not control for age as a potential confounder. If personality change would only occur due to biological maturation processes while some LE also normatively occur at a certain age, age effects could be easily mistaken for LE effects (Specht et al. 2011). As some LE only occur once or twice in a lifetime (if at all), researchers often compute aggregations of LE (e.g., by life domain or valence; Rakhshani et al. 2021; Specht et al. 2011; Wundrack et al. 2021) to investigate LE effects, which comes with a loss of important information. One the other hand, we know almost nothing about effects of the repeated occurrence of LE (Dugan et al. 2024), which could either lead to an adaption to or to an accumulation of stressors (Luhmann and Eid 2009).

The most prevalent limitation, however, is the overwhelming reliance on self‐reports of personality (Bleidorn et al. 2020; De Vries et al. 2021; Jackson and Beck 2021; Lüdtke et al. 2011). Of the 89 samples included in the meta‐analyses of Bühler, Orth, et al. (2024), not a single sample involved other assessments of personality than self‐reports, which is particularly concerning given that self‐ and informant reports are not always in agreement in terms of personality change (Costa et al. 2019). With respect to personality assessment, it is also notable that studies seem to focus exclusively on the Big Five traits, although other promising taxonomies exist, such as the HEXACO model (Ashton and Lee 2009). Although HEXACO traits have not shown fundamentally different change patterns as compared to the Big Five (Ashton and Lee 2016), an extension of LE research on other personality taxonomies would strengthen the robustness of evidence in this field.

Another limitation of research to date is the narrow focus on mean‐level changes (Atherton et al. 2020; Jackson and Beck 2021), which can only be expected for highly normative LE that involve transparent and uniform behavioral demands (Bühler, Orth, et al. 2024; Neyer et al. 2014). LE which do not have a uniform effects may instead increase individual differences in personality traits while LE providing clear behavioral scripts may decrease trait variability (Jackson and Beck 2021). Moreover, interindividual differences in trait change have often been neglected (Haehner et al. 2022; Lüdtke et al. 2011). People can differ in their experience of LE or in their ability and willingness to recognize and adapt to new role demands (Bleidorn et al. 2020; Specht 2017). Individual differences in change patterns may explain why research to date continues to have difficulties producing replicable effects. The individual perception of LE is one of the most discussed moderators of LE effects, as it contains information about their individual causes, circumstances, and consequences (Haehner et al. 2022; Luhmann et al. 2021). However, the few studies conducted on this topic to date either focused on single traits (Neuroticism; Kandler and Ostendorf 2016) or on single LE (graduating from school and moving away from home; De Vries et al. 2021). Most recently, Haehner et al. (2023) found some evidence for event characteristics to moderate personality change following LE; however, the study covered a comparatively short time interval and focused on a small selection of negative events. To the best of our knowledge, no study to date investigated interindividual differences in the effects of LE on different trait change patterns including a wide variety of LE from different life domains while also considering their individual perception.

### 1.4 Aim of the Current Study

As the four reviews on effects of LE on personality change and the substantial limitations discussed in the literature suggest, more research is needed to clarify under which circumstances personality changes following LE, which LE are most important for personality trait change and which traits change the most after the occurrence of LE (Bleidorn et al. 2018). With our study, we aim to extend previous research on this topic in several ways to address specific limitations of former studies with respect to research design and analyses.^1^

To investigate the effects of LE on trait change, we used a prospective multirater study design with three measurement occasions at 2‐year intervals. Self‐ and informant reports are used as indicators of HEXACO personality traits to compare the two perspectives in terms of trait change following LE. LE were surveyed with a new inventory, the Critical Life Event Categories Scale (CLECS; Oeltjen et al. 2025), which captures broader, but homogenous LE categories to ensure that these events are experienced by a sufficiently large number of people and do not need to be aggregated. The new inventory also captures the individual perception of LE in terms of valence and controllability. To investigate different types of personality trait change in one comprehensive model, we used a modeling approach based on the revised Latent‐State–Trait (LST‐R) Theory. LST‐R models not only capture latent mean‐level changes but also changes in interindividual trait differences (trait variability) and situational variability (state‐residual variance) around the changing trait, while controlling for variance due to random error of measurement (Steyer et al. 2015). A recent extension of LST‐R models, MNLST modeling (Oeltjen et al. 2023), enables the definition of these change patterns as a function of external covariates to allow for interindividual differences in personality trait change trajectories. This modeling approach allows us to investigate the effects of LE on trait change while controlling for potential confounders and including potential moderators like age, gender, the individual perception of LE, and their repeated occurrence. The model does not require making any assumptions about the shape of trait change but flexibly allows for any kind of change trajectory to emerge. Moreover, we did not only consider self‐reports, but also used informant‐reports as measures of personality traits. This multirater approach allowed us to examine the robustness of trait change patterns following LE across different rater perspectives.

Using the described research design and methodology, we first aimed to investigate how personality traits change after the occurrence of LE. Specifically, we addressed the following Research Questions:

1.1 Which personality traits show significant changes following which LE when controlling for age and gender?
1.2 Do LE show the same effects on personality change based on self‐ and informant reports?

Since the normativity of LE might play an important role (especially with respect to mean‐level changes) and is often linked to a certain age, it is reasonable to assume that LE may have a stronger effect on personality change depending on the age at which they occur (Bühler, Mund, et al. 2024). Furthermore, some LE (e.g., parenthood) may affect the life of men and women differently. In fact, some former studies already found differential effects of LE for men and women (Bleidorn et al. 2018) and across age (Bühler, Mund, et al. 2024). We will thus not only control for age and gender but also include them as moderators of the effects of LE on personality trait change.

1.3 Do age or gender moderate the effects of LE on personality trait change?

Emerging research also suggests that the effects of LE on personality change may diminish or even increase over time (Atherton et al. 2020; Bühler, Orth, et al. 2024). We will thus not only investigate the effects of LE on trait change at the subsequent measurement occasion but also the lagged effects of LE occurring between time 1 and 2 on personality change at time 3.

1.4 Do personality trait changes following certain LE diminish or intensify over time?

Finally, LE effects may not only vary depending on the time interval between event occurrence and personality assessment; their effects may also be altered when the same event occurs repeatedly for the same person, either in the sense of an adaptation or in the sense of a sensitization (Luhmann and Eid 2009). A recent study of Dugan et al. (2024) found initial evidence for a stronger (cumulative) effect of the repeated occurrence of LE on personality trait change. However, for life satisfaction, Krämer et al. (2025) found attenuated effects for the repeated occurrence of certain events.

1.5 Are effects of certain LE altered with the repeated exposure to these events?

In addition to effects of the occurrence of LE, we will secondly investigate whether the individual perception of LE has an effect on personality trait change. As already explained above, the individual perception of LE could represent an important moderator of LE effects on personality trait change (Haehner et al. 2022; Rakhshani et al. 2021). Different event characteristics may even interact in predicting personality change (Rakhshani et al. 2021).

2.1 Does the individually perceived valence or controllability predict personality change following LE?
2.2 Do valence and controllability interact in the prediction of personality change following LE?

In the following, we will outline our research design and the conducted analyses in greater detail.

## 2 Methods

### 2.1 Study Design and Participants

We will investigate our research questions based on data stemming from the Study of Personality Architecture and Dynamics (SPeADy). SPeADy is a multirater longitudinal study which aims to investigate the different components of personality considering various sources of variability. We use data from the age group substudy, of which a detailed description can be found in Wiechers et al. (2023). Participants were recruited with the help of leaflets, promotions stands and media calls to obtain a balanced and heterogenous sample with respect to age (≥ 14 years), gender, family status, and education. All participants provided written consent in accordance with the Declaration of Helsinki and EU data protection regulations prior to participating in the surveys. The ethics committees of Bielefeld University and Medical School Hamburg reviewed and approved SPeADy.

Personality traits, various self‐related concepts and environmental conditions were surveyed at three measurement occasions over 4 years, for each participant approximately 2 years apart (wave 1: 2016–2018, wave 2: 2018–2020 and wave 3: 2020–2022). The occurrence of LE was assessed retrospectively for targets at the second and third measurement occasions with respect to the previous time interval. Participants were asked for contact information of up to three informants, who were subsequently invited to participate in the study. The informant questionnaire comprised all constructs of the self‐report version (except for LE). In each assessment wave, participants could nominate the former or new informants.

In the first wave, *N* = 2405 people took part, *N* = 1504 in the second and *N* = 1154 in the third. To compensate for dropouts, new participants were recruited for the second and third wave. As we were interested in individual differences in trait change over different time intervals relative to time 1, only participants who took part in all three assessment waves (*N* = 623) were included. When they entered the study, the target persons were between 14 and 86 years old (*M* = 45.1, SD = 17.1), 64.8% female, and well educated (62.2% had at least a high school degree). The up to three informants per target of all three assessment waves were also predominantly female with a mean age between 40.7 (SD = 16.2) at time 1 and 45.0 (SD = 17.3) years at time 3. Most of the informants knew the targets very well or well and were acquainted with the targets for between 21.2 (SD = 14.3) and 23.9 (SD = 15.7) years on average. Details can be found in Appendix Table A1.

### 2.2 Measures

#### 2.2.1 Personality Traits

Personality traits were measured with the German version of the HEXACO‐60 (Ashton and Lee 2009; German version: Moshagen et al. 2014). The inventory involves six personality domains (Honesty‐Humility, Emotionality, Extraversion, Agreeableness, Conscientiousness and Openness) each captured with 10 items. Items (e.g., Extraversion: “On most days, I feel cheerful and optimistic.”) were answered on a 5‐point scale from 1 (“strongly disagree”) to 5 (“strongly agree”). Negatively keyed items were reversed for the statistical analyses such that higher values indicate higher scores on the above listed personality domains. Reliabilities of self‐ and informant‐reports at all three measurement occasions (all ≥ 0.70) can be found in Table A2.

#### 2.2.2 Life Events

LE were assessed with the Critical Life Event Categories Scale (CLECS, Oeltjen et al. 2025). The inventory captures 21 broader but homogenous LE categories from five life domains, the occupational/financial, social, housing, health domain and individual domain. The aim of the more general formulations of the LE (e.g., “Breakup/loss of a serious relationship”) is to ensure that these event categories are experienced by a sufficiently large number of people so that a subsequent aggregation of LE is no longer necessary. For each LE category, the individually perceived valence is assessed on a 7‐point scale from 1 (“very negative”) to 7 (“very positive”) and the individually perceived controllability is rated on 3‐point scale from 1 (“uncontrollable”) to 3 (“controllable”). To ease the interpretation, valence and controllability ratings were recoded from −3 to 3 and from—1 to 1, respectively, such that 0 represents the neutral value. A list of all LE categories and their abbreviations can be found in Table 2 and more detailed in Appendix Table B1, for frequencies and descriptive statistics of LE perceptions see Table B2.

**TABLE 2:** List of life events.

Abbreviation	Life event
Occ+	Improvement in educational/occupational situation
Occ–	Deterioration in educational/occupational situation
Fin+	Improvement in financial situation
Fin‐	Deterioration in financial situation
NewE	Start of a new educational/training/occupational stage
Degr	Achievement of an important degree
Relo	Relocation
House	Considerable change in domestic/family situation
Confl	Serious conflict with other people
Rel‐	Breaking up/loss of a close relationship/partnership
Rel+	Entering a close relationship/partnership
Child	Pregnancy/birth of own child/adoption of a child
Soc+	More social activities during leisure time
Soc‐	Fewer social activities during leisure time
IllnO	Severe physical or mental illness, injury of a loved one
PhyS	Severe own physical illness or injury
PsyS	Own mental illness or addiction
Succ	Significant personal success
Fail	Significant personal failure
Crime	Experiencing a crime
Catas	Experiencing a life‐threatening situation

### 2.3 Statistical Analysis

To investigate the impact of LE on personality trait change, we run MNLST models. This approach builds on Bayesian estimation techniques with Monte Carlo Markov Chain (MCMC) sampling as implemented in JAGS (Plummer 2003). In a MNLST model, trait change and variability parameters of an LST‐R model are regressed on external (time‐varying or time‐invariant) covariates to allow for and explain interindividual differences in trait change and situational variability. In LST‐R Theory, the trait $\xi_{\mathrm{it}}$ (of indicator *i* at time *t* > 1) is a positive linear function of the trait at time 1, $\xi_{i1}$:

(1) $$\xi_{\mathrm{it}}=\alpha_{\mathrm{it}}+\lambda_{\mathrm{it}}\xi_{i1}\;\text{for}\;t>1$$

The shape of the trait change trajectory is, on the one hand, captured in the intercepts $\alpha_{\mathrm{it}}$ (for *t* > 1), which reflect the mean shift of trait values across time. To obtain additive trait change parameters $\Delta_{\mathrm{it}}$ (i.e., mean‐level changes with respect to time 1), initial intercepts ($\alpha_{i1}$) are included in model equations of parameters $\alpha_{\mathrm{it}}$ for *t* > 1 (see Oeltjen et al. 2023, for details). On the other hand, trait factor loadings $\lambda_{\mathrm{it}}$ (for *t* > 1), that is, multiplicative change parameters, indicate the increase ($\lambda_{\mathrm{it}}$ > 1) or decrease ($\lambda_{\mathrm{it}}$ < 1) of interindividual differences in the latent trait in relation to the first measurement occasion, that is, changes in trait variance.

In a first step, before the MNLST analyses, we run unmoderated LST‐R models for each personality dimension to evaluate the fit of the base model. As the MNLST model, which allows parameters to vary across values of the external covariates, is always more flexible than the corresponding (unmoderated) base model, it represents the lower bound for model fit. LST‐R models were specified across three measurement occasions with aggregated (mean) scores of self‐ and informant reports as indicators and indicator‐specific trait variables (see Appendix Figure C1). This allows a differentiated investigation of trait change from the self‐ and informant perspective. All factor means were fixed to zero, and the first loading per trait factor was fixed to one. As base models of all personality traits indicated the absence of cross‐rater state‐residual variables, with situational variability not being significantly different from 0, they were set to 0 for all subsequent analyses.

In the next step, we included covariates in MNLST models to explain interindividual differences in trait change. In the first series of models, LE were included as time‐varying predictors of additive and multiplicative trait change parameters at the second and third measurement occasion. Their effects were set equal across time. We also included the lagged effects of LE occurring between time 1 and time 2 on trait change at time 3 to investigate whether the effect increases or decreases with time. Gender and age (centered) at time 1 were included as time‐invariant covariates of trait change and initial values ($\alpha_{i1}$). We also tested interactions (LE × age and LE × gender) to investigate whether the effect of the occurrence of LE depends on age or gender. To examine the effect of the repeated occurrence of LE (i.e., their cumulative effects), we included the interaction between event occurrence at time 2 and event occurrence at time 3 on trait change at time 3. Regression equations of MNLST models for all moderated parameters are outlined in Appendix D. We fitted one MNLST model per combination of LE and personality trait, resulting in a total of 126 models.

In a second series of models, we investigated the effect of the individual perception of LE on trait change (for those who experienced them). We only included the LE, which occurred frequently enough (with a minimum of 120 participants providing data on LE perceptions at both measurement occasions) to adequately investigate the effect of their individual perception on personality trait change (see Oeltjen et al. 2023, for a simulation study on the MNLST approach). The individual perceptions of LE in terms of valence and controllability were included as time‐varying covariates of trait change parameters at the second and third measurement occasions. We also tested the interaction between both event characteristics to investigate whether the effect of valence becomes more positive or negative with increased controllability (and vice versa). We again estimated separate models for each LE‐trait combination, which resulted in an additional 60 models.

Significance of trait change parameters and covariate effects was evaluated using 95% credibility intervals. All analyses were run in R (R Core Team 2023), annotated code is provided in the online material (https://osf.io/uqwab). Base LST‐R models were fit using the maximum likelihood robust estimator implemented in lavaan (v0.6–4; Rosseel 2012). MNLST analyses were carried out using rjags (Plummer 2023). All unconstrained parameters were modeled using weakly informative priors (see Appendix E for details). Estimation was conducted by running three MCMC chains with 12,000 iterations each; 3000 were discarded as burn‐in. Convergence of MCMC chains was evaluated by visually inspecting autocorrelation and trace plots (see online material, https://osf.io/rbzfn). MCMC chains converged well with only very few exceptions for certain parameters in some of the models.

## 3 Results

Unmoderated base models of all personality traits fit the data well, with CFI > 0.990, TLI > 0.982, RMSEA < 0.057 and SRMR < 0.021 (see Appendix Table C1 for details). Correlations between method‐specific trait factors ranged from 0.53 (Honesty‐Humility) to 0.78 (Openness). No substantial additive or multiplicative change was observed in the unmoderated LST models (i.e., over the entire sample). For Honesty‐Humility and Conscientiousness informants reported systematically higher values while levels of Agreeableness and Openness were rated higher by targets. The differences, however, were small.

### 3.1 Occurrence of LE

Significant effects of the LE occurrence on additive and multiplicative trait change (i.e., mean‐level changes and changes in trait variance) in the six personality domains are summarized in Tables 3 and 4, respectively. The $b_{3}$ coefficients indicate effects of the respective LE on trait change at the subsequent measurement occasion (e.g., effect of a life event occurring between time 1 and 2 on trait change at time 2). Note that trait change for t>1 is always defined in relation to the first measurement occasion. $b_{4}$ and $b_{5}$ indicate, whether and how these effects depend on gender and age, respectively. $b_{6}$ indicates long‐term effects of LE, that is, how an event taking place between time 1 and 2 affects trait change at time 3. $b_{7}$ represents the interaction between event occurrence in the first time interval (between time 1 and 2) and in the second time interval (between time 2 and 3) on trait change at time 3, that is, the effect of the repeated exposure to a LE (i.e., cumulative effect). For a small number of parameters, chains did not converge properly, so they have to be interpreted with caution and are marked with an asterisk. Detailed results of all models fit to investigate the effects of the occurrence of LE on personality traits can be found in the online material (https://osf.io/cwahj).

**TABLE 3:** Effects of the occurrence of LE on additive trait‐change.

LE	Honesty‐Humility					Emotionality					Extraversion					Agreeableness					Conscientiousness					Openness				
	$b_{3}$	$b_{4}$	$b_{5}$	$b_{6}$	$b_{7}$	$b_{3}$	$b_{4}$	$b_{5}$	$b_{6}$	$b_{7}$	$b_{3}$	$b_{4}$	$b_{5}$	$b_{6}$	$b_{7}$	$b_{3}$	$b_{4}$	$b_{5}$	$b_{6}$	$b_{7}$	$b_{3}$	$b_{4}$	$b_{5}$	$b_{6}$	$b_{7}$	$b_{3}$	$b_{4}$	$b_{5}$	$b_{6}$	$b_{7}$
	Occupational and Financial Domain																													
Occ+ SR								+			+		—																	
Occ+ IR																													−	
Occ− SR			+																											
Occ− IR													−																	
Fin+ SR						−			−					+										+						
Fin+ IR																							−							
Fin− SR		+							−							−									+					
Fin− IR																														
NewE SR							−																							
NewE IR																			−							+				
Degr SR																														
Degr IR					+			+	−	+					−															
	Housing Domain																													
Relo SR														−									+							+
Relo IR																														
House SR																		+												+
House IR		−																												
	Social Domain																													
Confl SR									−											+				−						
Confl IR																												−		
Rel− SR								−								+							−						+	
Rel− IR																								+						
Rel+ SR										+										**−**										
Rel+ IR								+										−	+	**−**	−	+		+						
Child SR																										−			−	
Child IR								+					−								−							−		
Soc+ SR													−										+			+				
Soc+ IR								+																				+		
Soc− SR											−		+																	−
Soc− IR																					+									
	Health Domain																													
IllnO SR																														
IllnO IR						−				+			+																	
PhyS SR			−								−																			
PhyS IR													+		−													+		
PsyS SR											−					**−**		+				−								
PsyS IR																**−**					−									
	Individual Domain																													
Succ SR	−																													
Succ IR																		−												
Fail SR											**−**								−			−								
Fail IR					−						**−**		−															−		
Crime SR		+		−																			+			+				
Crime IR																													+	
Catas SR														−				+	−			−								
Catas IR				+	−	−									−		+													

*Note:* $b_{3}$: Effect of the occurrence of the respective LE on trait change parameter at the subsequent measurement occasion, $b_{4}$: Interaction effect between event occurrence and gender (with 0 = female and 1 = male), $b_{5}$: Interaction effect between event occurrence and age (centered). $b_{6}$: Long‐term effect of the occurrence of the respective LE between time 1 and 2 on trait change parameter at time 3, $b_{7}$: Effect of the repeated exposure to the respective LE. SR: self‐report, IR: informant‐report. For abbreviations of LE see Table 2. Significant positive effects are denoted with +, significant negative effects are denoted with −. LE effects consistent across self‐ and informant reports are printed in bold type.

**TABLE 4:** Effects of the occurrence of LE on multiplicative trait‐change.

LE	Honesty‐Humility					Emotionality					Extraversion					Agreeableness					Conscientiousness					Openness				
	$b_{3}$	$b_{4}$	$b_{5}$	$b_{6}$	$b_{7}$	$b_{3}$	$b_{4}$	$b_{5}$	$b_{6}$	$b_{7}$	$b_{3}$	$b_{4}$	$b_{5}$	$b_{6}$	$b_{7}$	$b_{3}$	$b_{4}$	$b_{5}$	$b_{6}$	$b_{7}$	$b_{3}$	$b_{4}$	$b_{5}$	$b_{6}$	$b_{7}$	$b_{3}$	$b_{4}$	$b_{5}$	$b_{6}$	$b_{7}$
	Occupational and Financial Domain																													
Occ+ SR											−	+	−																	
Occ+ IR																														
Occ− SR															−*															
Occ− IR																														
Fin+ SR	+										−	+		−	+															
Fin+ IR																														
Fin− SR						+	−																							
Fin− IR																							+							
NewE SR												+	−		−															
NewE IR																											+	−		
Degr SR												+																		
Degr IR								+																						
	Housing Domain																													
Relo SR								+																						
Relo IR																														
House SR																													−	+
House IR								+				−																		
	Social Domain																													
Confl SR											−	+												+	**−**					
Confl IR					−																+	−			**−**				+	
Rel− SR						+																								
Rel− IR					−*			+													+									
Rel+ SR																								−	+					
Rel+ IR																													−	
Child SR																			−*											
Child IR			−									−*		−*					−*							−*			+*	
Soc+ SR																						+	−							
Soc+ IR			−																								−			
Soc− SR												+			+										+					
Soc− IR			+		+																							−		
	Health Domain																													
IllnO SR																														
IllnO IR			−																			−								
PhyS SR																		+												
PhyS IR		−*		+																										
PsyS SR																	+		+									+	+	−
PsyS IR		−																												
	Individual Domain																													
Succ SR											−	+																		
Succ IR								+																						
Fail SR			**+**									+				+				−		+			+					
Fail IR	+		**+**																−*				+	−						
Crime SR							+																		**−**					
Crime IR					−*		−*						+												−*					
Catas SR																														
Catas IR				−*					−*																					+*

*Note:* $b_{3}$: Effect of the occurrence of the respective LE on trait change parameter at the subsequent measurement occasion, $b_{4}$: Interaction effect between event occurrence and gender (with 0 = female and 1 = male), $b_{5}$: Interaction effect between event occurrence and age (centered). $b_{6}$: Long‐term effect of the occurrence of the respective LE between time 1 and 2 on trait change parameter at time 3, $b_{7}$: Effect of the repeated exposure to the respective LE. SR: self‐report, IR: informant‐report. For abbreviations of LE see Table 2. Significant positive effects are denoted with +, significant negative effects are denoted with −. LE effects consistent across self‐ and informant reports are printed in bold type. Effects for which the estimator did not converge properly are marked with an Asterisk.

#### 3.1.1 Honesty‐Humility

Additive change in Honesty‐Humility was comparatively independent of the occurrence of LE and primarily observed following events from the individual life domain. For example, a decrease in (self‐reported) Honesty‐Humility was observed following the experience of a Success (*Succ*). In terms of multiplicative change, particularly the repeated occurrence of events from the social domain was associated with changes in interindividual trait differences in Honesty‐Humility as, for instance, people tend to get more similar following the repeated experience of a conflict (*Confl*). Most effects were only observed in informant reports; however, both self‐ and informant reports indicated that the positive effect of experiencing a Failure (*Fail*) on multiplicative change increased with age.

#### 3.1.2 Emotionality

Particularly events from the occupational domain (e.g., achievement of a degree, *Degr*) were associated with a long‐term decrease in Emotionality. The negative effect of experiencing an improvement in one's financial situation (*Fin+*) on Emotionality also appeared in the short term and seems to persist over time. The impact of events from the social domain on Emotionality was closely linked to age. Only a few effects on multiplicative change in Emotionality were found. The experience of a deterioration of the financial situation (*Fin–*) was associated with an increase in Emotionality trait variance, which was less pronounced for men.

#### 3.1.3 Extraversion

Extraversion was one of the traits that showed the most changes following LE. Both self‐ and informant‐reports indicated a decrease in Extraversion following the experience of a failure (*Fail*), which was even stronger for older individuals. Also, events from the health domain, namely the experience of a mental (*PsyS*) or physical (*PhyS*) illness, were associated with a decrease in this trait. The experience of an improvement in the occupational situation (*Occ+*) was associated with both additive and multiplicative change in Extraversion, that is, people tend to show an increase in Extraversion and at the same time get more similar (i.e., show a decrease in trait variance) following this event. The same effect was observed (in the long term) following the experience of an improvement in one's financial situation (*Fin+*). However, all these effects were only observed in self‐reports. LE effects on additive change in Extraversion particularly depended on the moderator age, while effects on multiplicative change appeared to depend on the moderator gender. Particularly gain‐based events of the occupational domain (and also the experience of a success, *Succ*) were associated with a decrease in trait variance, which was less pronounced for men, that is, especially women tend to get more similar following these events.

#### 3.1.4 Agreeableness

With respect to additive change, both self‐ and informant reports indicated a decrease in Agreeableness after experiencing a mental illness (*PsyS*), an effect that was less pronounced for older individuals. Also, the repeated experience of entering a close relationship (*Rel+*) was robustly (across personality assessment methods) associated with a decrease in Agreeableness. Only a few LE effects on multiplicative change in Agreeableness emerged. The experience of a Failure (*Fail*), for example, was associated with an increase in trait variance, but with the repeated experience of this event, the effect was reversed.

#### 3.1.5 Conscientiousness

Additive change in Conscientiousness was particularly associated with events from the social domain. For example, entering a close relationship (*Rel+*) and birth of a child (*Child*) were both followed by a decrease in this trait. However, these effects were almost exclusively observed in informant‐reports of personality, and the negative effect of entering a close relationship was reversed in the long term. The experience of a conflict (*Confl*) was (in the long term) associated with a decrease in Conscientiousness and, at the same time, with an increase in interindividual trait differences. Moreover, interindividual differences in Conscientiousness were particularly associated with the repeated occurrence of certain LE from the social and individual life domains. For the repeated experience of a conflict (*Confl*), both self‐ and informant‐reports indicated a subsequent decrease in trait variance.

#### 3.1.6 Openness

With respect to additive change, the birth of a child (*Child*) was associated with a decrease in Openness, which also persisted in the long term. Moreover, both events from the housing domain (relocation, *Relo*, and change in the domestic situation, *House*) were followed by an increase in Openness, but only with a repeated experience of these events. In terms of multiplicative change, Openness primarily showed long‐term changes following LE. For example, the experience of a change in the domestic situation (*House*) was associated with a long‐term increase in trait variance; however, with the repeated experience of this event, the effect was reversed and no effect was robustly found across assessment methods.

### 3.2 Individual Perception of LE

We investigated the effects of the individual perception of LE on personality trait change for the ten most frequently occurring LE. Effects of the perceived valence ($b_{1}$) and controllability ($b_{2}$) of these LE, as well as their interaction ($b_{3}$), on additive and multiplicative trait change are shown in Tables 5 and 6, respectively. Detailed results can be found in the online material (https://osf.io/zsyre).

**TABLE 5:** Effects of the individual perception of LE on additive trait‐change.

	Honesty‐Humility			Emotionality			Extraversion			Agreeableness			Conscientiousness			Openness		
	$b_{1}$	$b_{2}$	$b_{3}$	$b_{1}$	$b_{2}$	$b_{3}$	$b_{1}$	$b_{2}$	$b_{3}$	$b_{1}$	$b_{2}$	$b_{3}$	$b_{1}$	$b_{2}$	$b_{3}$	$b_{1}$	$b_{2}$	$b_{3}$
	Occupational and financial domain																	
Occ+ SR													+			+	**+**	**−**
Occ+ IR							+										**+**	**−**
Fin+ SR							+									+		
Fin+ IR																		
NewE SR							**+**						**+**					
NewE IR							**+**											
Degr SR			−										+					
Degr IR							+				+							−
	Housing domain																	
Relo SR					+	−												
Relo IR		+	−								+	−			−			
House SR				−						+	−							
House IR								+	−					−				
	Social domain																	
Confl SR					−							+						
Confl IR																		
Soc+ SR																		
Soc+ IR																		
	Health domain																	
IllnO SR																		
IllnO IR																		
	Individual domain																	
Succ SR											+						−	+
Succ IR					+	−												

*Note:* $b_{1}$: Effect of the perceived valance of the respective LE on trait change, $b_{2}$: Effect of the perceived controllability of the respective LE on trait change, $b_{3}$: Effect of the valance × controllability interaction on trait change. SR: self‐report, IR: informant‐report. For abbreviations of LE see Table 2. Significant positive effects: +, Significant negative effects: −. Effects consistent across self‐ and informant reports are printed in bold type.

**TABLE 6:** Effects of the individual perception of LE on multiplicative trait‐change.

	Honesty‐Humility			Emotionality			Extraversion			Agreeableness			Conscientiousness			Openness		
	$b_{1}$	$b_{2}$	$b_{3}$	$b_{1}$	$b_{2}$	$b_{3}$	$b_{1}$	$b_{2}$	$b_{3}$	$b_{1}$	$b_{2}$	$b_{3}$	$b_{1}$	$b_{2}$	$b_{3}$	$b_{1}$	$b_{2}$	$b_{3}$
	Occupational and financial domain																	
Occ+ SR										+								
Occ+ IR																		
Fin+ SR															−			
Fin+ IR						+												
NewE SR			−															
NewE IR			+													−		
Degr SR																		
Degr IR									+									
	Housing domain																	
Relo SR				+														
Relo IR																		
House SR																		
House IR														+				
	Social domain																	
Confl SR																	−	
Confl IR		+				+												
Soc+ SR							−	−	+									
Soc+ IR																		
	Health domain																	
IllnO SR													−				−	
IllnO IR															−			
	Individual domain																	
Succ SR					+													
Succ IR		−	+															

*Note:* $b_{1}$: Effect of the perceived valance of the respective LE on trait change, $b_{2}$: Effect of the perceived controllability of the respective LE on trait change, $b_{3}$: Effect of the valance × controllability interaction on trait change. SR: self‐report, IR: informant‐report. For abbreviations of LE see Table 2. Significant positive effects: +, Significant negative effects: −.

The perceived valence of LE (primarily of the occupational domain) showed mostly positive effects on additive trait change in Extraversion, Conscientiousness, and Openness; hence, a more positive evaluation was associated with a steeper increase and a more negative evaluation with a steeper decrease in these traits. Emotionality decreased with a more positive evaluation of an experienced change in the domestic situation (*House*). However, only the effect of the start of a new occupational stage (*NewE*) on additive change in Extraversion was found for both self‐ and informant reports.

The perceived controllability of LE was particularly relevant for events from the housing and individual domain. For example, an increased controllability of experiencing a Relocation (*Relo*) was associated with a steeper increase in Honesty‐Humility, Emotionality, and Agreeableness. It is worth noting that in most cases the effect of controllability was attenuated with a more positive evaluation of the event. That means, for an event being perceived as controllable and positive at the same time has no amplifying multiplicative effect on additive trait change, but rather attenuates the main effect. With few exceptions, the overwhelming number of effects were again only found for either self‐ or informant reports. Only the effect of controllability with regard to an improvement in one's occupational situation (*Occ+*) on Openness appeared robustly across assessment methods. Overall, only few effects of the individual perception of LE on multiplicative change were found. Conversely to effects on additive change, no clear effect patterns emerged, and no effect was confirmed across self‐ and informant‐reports.

## 4 Discussion

With this paper, we extended the current state of research on effects of LE on personality trait change in several ways. The effects of the occurrence of 21 LE from 5 life domains on different types of trait change were investigated under consideration of different moderators, such as gender, age, the time‐lag between event occurrence and personality change, and the repeated occurrence of LE. Moreover, we included the individual perceptions of LE in terms of valence and controllability as covariates of trait change patterns. Using data from a longitudinal multirater study, we were able to comparatively examine these effects in self‐ and informant‐reports. Several key findings can be derived:

First, in terms of additive (i.e., mean‐level) change, all HEXACO personality traits showed an almost equal number of LE effects, with the exception Honesty‐humility, for which only a few LE effects (primarily from the individual domain) were found. Overall, most effects were found for events from the social and individual life domain, whereby LE from the social domain seem to be particularly relevant for Conscientiousness and Openness. Events from the housing domain showed the fewest LE effects, although their repeated occurrence was associated with increases in Openness. Various explanations have been offered to explain why certain life events may be followed by more pronounced mean‐level changes in certain traits than others. Social investment theory (Roberts et al. 2005), for example, suggest that those LE involving new social roles and transparent behavioral demands with strong reward and punishment structures have the greatest potential to induce mean‐level changes. However, the events to which this applies (e.g., birth of a child) did not show particularly many effects in this study. In the most recent review and meta‐analysis by Bühler, Orth and colleagues (2024) more significant effects for events from the work than from the social domain were found. This contrasts with our results, which indicate the most effects for events from the social and individual domain. However, a substantial number of LE effects from the social domain in our study were only found for informant‐reports. Considering that the meta‐analysis of Bühler, Orth, et al. (2024) exclusively relied on self‐report studies, it seems plausible that some important effects from the social domain may have been missed there. Beyond general patterns, effects of specific LE on mean‐level changes in personality as found in the reviews of Specht (2017), Bleidorn et al. (2018), Bühler, Orth, et al. (2024), and Haehner et al. (2025), see Table 1 were only partially replicated in this study. Of the five effects that were at least confirmed by three of the reviews, only two were found in our study: the positive effects of divorce (in our study: loss of a close relationship) on Agreeableness and Openness. However, the comparability of our results to those of previous studies is limited as this study involves several novel aspects (e.g., analytic approach, LE measure, HEXACO instead of Big Five model).

Second, in terms of multiplicative change (i.e., changes in trait variance), Extraversion and Conscientiousness changed the most following LE. Multiplicative change in Extraversion was particularly associated with LE from the occupational domain while trait variance in Conscientiousness was primarily followed by LE from the social and individual domain, particularly by their repeated occurrence. Again, events from the social domain showed the most effects, while LE from the Housing domain showed almost no effects. In the only study we found to extensively examine changes in trait variance following LE (Jackson and Beck 2021), trait variance after the event was almost always lower (except for LE from the health domain, which were associated with higher trait variance). The authors concluded that LE may constrain trait variance making people more similar. Overall, we did not find such a clear pattern: LE effects on multiplicative change depended on the individual combination of LE and trait and in direct comparison we even found slightly more positive effects on trait variance. Only trait variance in Extraversion indeed seems to decrease following LE, especially for women following gain‐based events from the occupational domain.

Third, most LE were followed by either additive or multiplicative trait change. Only for a few LE both types of change emerged at the same time. Experiencing an improvement of the occupational situation, for example, was followed by an upshift in (self‐reported) Extraversion combined with a decrease in trait variance, while conversely, experiencing a serious conflict was associated with a long‐term downshift in Conscientiousness but increased trait variance. However, this combined occurrence of additive and multiplicative change following LE was rare. With very few exceptions, multiplicative change was found for LE‐trait‐combinations that did not indicate additive change and vice versa. This fits with the theory that some LE may reinforce individual personality tendencies *instead* of inducing mean level changes (Jackson and Beck 2021). Overall, we found more effects on additive than on multiplicative personality change. However, this might be a methodological issue as effects on multiplicative change are harder to detect and require larger sample sizes as compared to effects on additive change (Oeltjen et al. 2023).

Fourth, overall, age was a more important moderator of LE effects on additive change in personality traits than gender, except for effects on additive change in Conscientiousness, which also showed multiple interactions with gender. For multiplicative change, this pattern was a little more ambiguous. Notably, several interactions between LE and gender were found to be associated with changes in Extraversion, indicating a steeper decrease in trait variance for women. In their review, Bleidorn et al. (2018) also considered interactions between LE and gender; however, the reported effects were not confirmed in our study. Instead, various other interactions with gender emerged. Bühler, Mund, et al. (2024) investigated age as a moderator of relationship event effects, but as opposed to our results, the authors report almost no significant interactions between age and the occurrence of relationship events on personality trait change. The only significant interaction effect on Conscientiousness found between linear age and beginning a relationship was not replicated in our study.

Fifth, with few exceptions, as for example the negative effect of experiencing an improvement of the financial situation on additive change in Emotionality or the negative effect of the birth of a child on Openness, most LE effects depended on the considered time interval. That is, they occurred either in the short term (i.e., at the subsequent measurement occasion) or in the long term (LE occurring between time 1 and 2 affecting trait change at time 3). In one case (the effect of Entering a close relationship on additive change in Conscientiousness) the short‐term effect was even reversed in the long term. Events from the health domain indicated only short term‐effects on additive trait change. On the contrary, multiplicative change in Openness primarily occurred in the long term. Our findings are contrary to results of Atherton et al. (2020) who found that effects generally tended to be smaller with longer time lags. In our study, this seems to depend on the specific LE‐Trait combination. Overall, we found about as many short‐term as long‐term effects. Bühler, Orth, et al. (2024) found a more differentiated pattern with gain‐based events from the love domain showing larger short‐term and loss‐based events showing larger long‐term effects. No such systematic pattern is evident in our findings. However, the exact timing of events was not assessed in our study which hinders a more fine‐grained analysis.

Sixth, in several cases only the repeated exposure to a specific LE (i.e., the cumulative effect of LE) was associated with additive or multiplicative trait change. For example, only the repeated experience of events from the Housing domain (relocation and change in the domestic situation) was associated with an increase in Openness at time 3. Also, multiplicative change in Conscientiousness seemed to depend on the repeated occurrence of certain LE, particularly from the social and individual domain. This fits with the findings of Dugan et al. (2024) which suggest potentially cumulative effects of the repeated occurrence of LE. However, in our study, we also found some LE effects to be diminished with the repeated occurrence of the respective LE. This indicates that the repeated occurrence of a specific event can indeed alter its effect, but whether this happens in terms of an accumulation or adaptation depends on the particular combination of LE and trait.

Seventh, valence and controllability predicted the amount of additive trait change in response to LE in several cases. Valence was particularly predictive of additive trait change in Extraversion, Conscientiousness, and Openness following LE from the occupational domain, where a more negative evaluation of the respective LE was associated with a steeper decrease in the respective trait. This fits with findings of previous studies suggesting that positive LE are associated with increases in Extraversion, Openness, Agreeableness, and Conscientiousness and Emotional Stability while experiencing negative events had the contrary effects (De Vries et al. 2021; Kandler and Ostendorf 2016; Specht 2017). Controllability primarily predicted additive change after events from the housing domain. It is worth noting that the mere occurrence of LE of the housing domain was hardly predictive of trait change but their individual perception showed numerous effects, particularly on additive change. This is in line with previous findings suggesting that the effects of the occurrence and perception of events can differ (Haehner et al. 2022). In fact, the effects of events that do not induce mean‐level changes may particularly depend on the individual circumstances, which are reflected in event characteristics. While former studies (e.g., Haehner et al. 2022; Luhmann et al. 2021) suggest that valence in particular might be a central property of LE, in our study both event characteristics were about equally relevant for the prediction of trait change, but for different LE‐trait combinations.

Eighth, with very few exceptions, such as the negative effect of experiencing a failure on additive change in Extraversion or the negative effect of experiencing a mental illness on Agreeableness, the overwhelming number of effects were specific to either self‐ or informant‐reports, although the correlations between rater‐specific trait factors were high overall. Interestingly, effects on additive change in Conscientiousness, particularly of events from the social domain, were predominantly found for informant‐reports. The same was true for multiplicative change in Honesty‐Humility and Openness, again, particularly following events from the social domain. Overall, these findings question the robustness of LE effects and their generalizability across personality assessment methods. If these results can be replicated, it would imply that previous studies, which are based almost exclusively on self‐reports (Bühler, Orth, et al. 2024), may have only revealed the self‐report‐specific part of the LE effects.

### 4.1 Limitations

The present study involves several novel aspects, such as the application of an advanced modeling approach, the inclusion of informant‐reports in addition to self‐ratings, the use of the HEXACO instead of the Big Five personality model and the consideration of various important moderators (especially event characteristics). This can be considered a strength but also a limitation, as it limits the comparability of our results to findings of previous studies. The HEXACO personality model, for example, shows substantial conceptual differences to the Big Five model, particularly with respect to the dimensions Emotionality, Agreeableness, and Honesty‐Humility (Thielmann et al. 2022). In accordance with most previous studies, this study is based on a WEIRD (Henrich et al. 2010) sample. Our findings may thus not generalize to other cultural contexts, in which other LE may normatively occur in other stages of life, with different frequencies, and, most importantly, with different evaluations of events. Studies on this topic carried out in other (non‐Western) parts of the world could lead to fundamentally different conclusions.

Another limitation of our data is that we were not able to examine state variability (i.e., situational deviations from the expected trait value for a person at time *t*) following LE. As already mentioned, most research to date focused on mean‐level changes while other types of change are usually neglected. Although we already extended this perspective by incorporating changes in trait variance following LE, it would have been interesting to also investigate whether certain LE are associated with stronger deviations from the expected trait level of a person or even with stronger situational fluctuations in the long term. Unfortunately, our data did not involve state‐residual variance for any of the six personality dimensions. This is an empirical finding that was beyond our control; however, we would have liked to take full advantage of the MNLST approach that would have allowed us to conduct such analyses. We strongly encourage researchers to consider analyses of state variability in more malleable characteristics (such as affective well‐being) following LE in future studies.

A limitation with respect to the research design is that the occurrence of LE was assessed retrospectively and that their exact timing and frequency was not assessed in our study. Participants were asked if a LE occurred at any point since the last assessment wave, hence, it was not specified whether the event occurred 5 days, 2 months, or 2 years ago. This only allowed for a very broad differentiation between “short‐term” (up to 2 years) and “long‐term” (2 to 4 years) effects. Previous studies suggest that effects may vary considerably as a function of the time interval between event occurrence and personality assessment (e.g., Atherton et al. 2020; Bühler, Orth, et al. 2024; Haehner et al. 2022). Thus, a more fine‐grained assessment of the timing would have been desirable. However, the retrospective assessment of the timing of events is not trivial, as individuals might differ in their perception of time‐lags and might not report them reliably. Similarly, the retrospective assessment of the occurrence of LE is prone to biases. Ecological momentary assessment studies, in which the occurrence of LE can be assessed in real‐time, may be the best solution for this problem and we strongly encourage researchers to include LE measures in respective study designs.

Finally, event characteristics were only assessed for LE that actually occurred. Therefore, we were only able to investigate effects of event characteristics of the most common life events. One solution for future studies could be to assess the hypothetical evaluation of LE of participants who did not experience the respective events. However, the assessment of evaluations of imagined LE can be difficult as people may have inaccurate beliefs about them (Haehner et al. 2022). Alternatively, it could be interesting to assess the evaluation of nonevents, that is, the evaluation of the nonoccurrence of an event (e.g., birth of a child) in a given time interval.

### 4.2 Future Directions

Like many studies before, the current study does indeed indicate that certain LE may be associated with changes in certain personality traits. Some of our findings are in line with findings of previous studies; however, many are not. This, to date, seems to be the only real consistency in research on personality trait change following LE. In most studies, a few effects emerge and some of them may even reappear occasionally, but almost no effect emerges with substantial robustness across different samples and contexts. The small overlap between findings of four reviews and meta‐analyses (see Table 1) proves this quite convincingly.

Our results suggest that most of the effects that emerge in the context of LE depend on the individual circumstances of the person or on methodological decisions of the researchers conducting the study. Only regarding the moderators covered in this study, we found that the amount of trait change following LE may depend on (a) the type of change under investigation, (b) the method used to measure personality, (c) the time passed since the event occurred, (d) whether the LE occurred once or repeatedly, (e) the composition of the sample with respect to age and gender, and (f) the individual evaluation of the life event in terms of valence and controllability. Given this volatility of LE effects, one can hardly expect to see much overlap between different studies that were not artificially paralleled with respect to these factors or explicitly consider them as moderators. These findings also suggest that the relationship between LE and personality change may be too complex and idiographic to be adequately described by simple nomothetic predictor‐and‐change relationships. We only included a small number of potential moderators, all of which were relevant to certain LE effects. In reality, LE effects may potentially depend on hundreds of moderators, from early childhood experiences to current resources and opportunities to future perspectives.

While some researchers have already recognized that we cannot expect LE to have uniform, population‐level effects, as they are extremely individual experiences that may initiate heterogenous change trajectories (Jackson and Wright 2024), others still aim to explain the observed heterogeneity in LE effects by including further covariates. In fact, most future directions discussed in the literature are essentially aimed at the consideration of additional moderators, such as the normativity of LE, the fit between the person and the event‐related changes (Bühler, Orth, et al. 2024), the size of the social network, perceptive skills (Specht et al. 2014), or the individual expectancy regarding the time after the LE (Hill and Allemand 2024). This list may be complemented by interviews or focus groups to explore further variables that might shape the individual response to LE (Weidmann et al. 2024).

The question is, however, up to which point the results of these analyses will be of practical use. First, a model of this complexity, containing such a large number of parameters (i.e., moderators), will not produce consistent estimates across studies, as it will always be overfitted to the sample at hand. Second, even if we find a consistent model of this complexity (which is unlikely), it would be too complex to actually serve as a basis for intervention measures or sociopolitical decisions. If we find that parenthood leads to a decrease of Emotional Stability in women, this could actually inform the design of support services for expectant mothers or assistance programs for re‐entry into working life. However, if we find that Emotional Stability decreases in young mothers, who got married 2 years ago, have twenty to thirty thousand dollars in their bank account and play tennis twice a week, this will hardly be of any practical use. Although traits might change in response to LE, these effects seem to depend on too many contextual factors to identify homogenous effects across studies that would be of societal relevance. We do not argue for abandoning this area of research, but to keep the question of practical benefit in mind when allocating resources to future studies. If research on the effects of LE on personality changes continues, it could benefit from a clear theoretical framework that outlines which LE might trigger changes in which personality traits under which circumstances, rather than combining all LE, traits, and potential moderators in an exploratory manner.

### 4.3 Conclusion

Like many former studies, we have shown that LE can indeed be associated with personality trait change. However, most of these effects are not consistent with previous research and depend on the method of personality assessment, the time lag between event occurrence and personality assessment, or the individual evaluation of the event. Although there are still many opportunities to further expand research on this topic, it is questionable whether these efforts will result in a stable model that is still of practical use.

## Author Contributions

L.O.: conceptualization, methodology, data curation, formal analysis, writing – original draft. C.K.: writing – review and editing, supervision.

## Funding

This work was supported by the Deutsche Forschungsgemeinschaft (Grant 272981829).

## Conflicts of Interest

The authors declare no conflicts of interest.

## Figures and Tables

**FIGURE C1:**
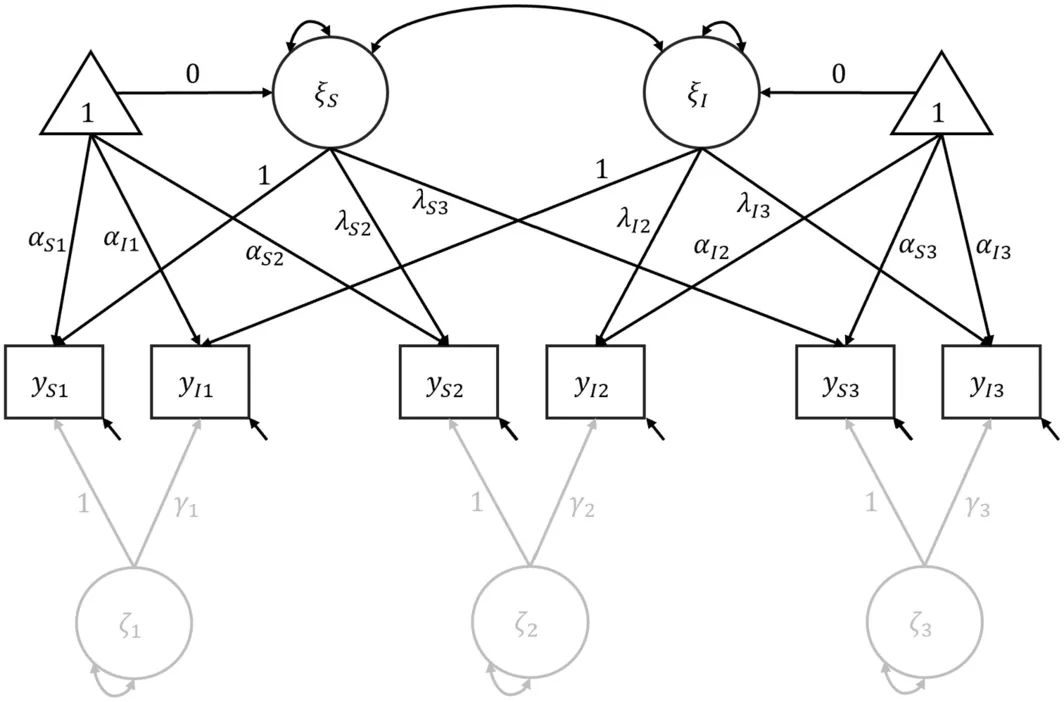
LST‐R base model with rater‐specific trait factors over three measurement occasions. 𝜉: Latent trait variable, 𝜁: Latent state‐residual variables at time *t*, 𝑦: Observed variables at time *t*, α: Intercepts at time *t*, 𝜆: Trait‐factor loadings at time *t*, 𝛾: State‐residual factor loadings at time *t*. *S*: Self‐reports, *I*: Informant‐reports. State‐residual factors are depicted in light gray as our data did not involve cross‐rater state‐residual variance for any of the six personality dimensions.

## Data Availability

Study materials have been uploaded to the Open Science Framework (https://osf.io/cwahj, https://osf.io/zsyre, https://osf.io/rbzfn). The analysis scripts can be downloaded via the following link: https://osf.io/uqwab. Access to the data can be requested here: http://www.speady.de/contact/?lang=en.

## Endnotes

^1^
An earlier version of this project has been preregistred (https://osf.io/8btqz). However, the preregistration was conducted in 2023, more than 1 year before the actual start of this project. Given the vibrant research in this field with many recent publications, our focus for this project has shifted in the meantime. Even though we have set the same high standards for a research process of scientific integrity for this article, we do not consider the article as preregistered in its current form.

## Appendix A Descriptive Statistics and Reliabilities

**TABLE A1:** Sociodemographic information of targets and informants.

Sociodemographic variable	Targets	Informants		
	Wave 1	Wave 1	Wave 2	Wave 3
Gender: % female	64.8	62.3	67.4	64.7
Age (in years): *M* (SD)	45.1 (17.1)	40.7 (16.2)	43.1 (17.0)	45.0 (17.3)
Family status (%)				
Unwed	35.5	31.9	33.8	38.3
Married	51.4	31.0	27.2	25.1
Divorced	10.1	36.5	38.4	36.1
Widowed	3.0	0.7	0.6	0.5
Education (%)				
School leaving certificate	4.3			
Secondary school certificate	18.8			
Polytechnic degree	8.7			
High school degree	20.1			
Bachelor's degree	7.4			
Master's degree	29.9			
Doctoral degree	4.8			
Others	2.2			
No school leaving certificate	3.9			
Closeness to target (%)				
Very poor		0.2	0.2	0.5
Poor		0.2	0	0
Medium		3.2	2.7	2.6
Good		29.6	23.7	27.8
Very good		66.7	73.3	69.0
Duration of relationship (in years): *M* (SD)		21.2 (14.3)	23.7 (14.5)	23.9 (15.7)

**TABLE A2:** Reliabilities of HEXACO traits.

Traits	Self‐reports			Informant‐reports		
	T1	T2	T3	T1	T2	T3
Honesty‐humility	0.70	0.73	0.72	0.75	0.77	0.76
Emotionality	0.75	0.78	0.79	0.81	0.82	0.80
Extraversion	0.81	0.80	0.79	0.82	0.80	0.79
Agreeableness	0.74	0.73	0.72	0.83	0.83	0.83
Conscientiousness	0.75	0.76	0.76	0.81	0.84	0.80
Openness	0.71	0.73	0.73	0.77	0.71	0.74

*Note:* Reliabilities: McDonald's Ω. T1–T3: Time 1 to Time 3.

## Appendix B Critical Life Events

**TABLE B1:** Items and abbreviations of the critical life event categories scale.

Abbreviation	English	German
Occ+	*Improvement in educational/occupational situation* (e.g., due to promotion, improved working conditions, improved relationship with colleagues/teachers/superiors)	*Verbesserung der schulischen/beruflichen Situation* (z. B. durch Beförderung, verbesserte Arbeitsbedingungen, verbessertes Verhältnis zu Kollegen/Lehrenden/Vorgesetzten)
Occ‐	*Deterioration in educational/occupational situation* (e.g., due to demotion, dismissal, poorer working conditions, deteriorated working atmosphere)	*Verschlechterung der schulischen/beruflichen Situation* (z. B. durch Zurückstufung, Kündigung, schlechtere Arbeitsbedingungen, verschlechtertes Arbeitsklima)
Fin+	Considerably more money available/*improvement in financial situation*	Wesentlich mehr Geld zur Verfügung/*Verbesserung der finanziellen Situation*
Fin‐	Considerably less money available/*deterioration in financial situation*	Wesentlich weniger Geld zur Verfügung/*Verschlechterung der finanziellen Situation*
NewE	*Start of a new educational/training/occupational stage* (e.g., change of school/studies/workplace, start of training/studies/professional life)	*Beginn eines neuen schulischen/ausbildungsbezogenen/beruflichen Abschnittes* (z. B. Wechsel von Schule/Studium/Arbeitsplatz, Beginn von Ausbildung/Studium/Berufsleben)
Degr	*Achievement of an important degree* (e.g., graduation from school/training/university, certificates and further qualifications, Ph.D., licensure, retirement)	*Erreichen eines wichtigen Abschlusses* (z. B. Schul‐/Ausbildungs‐/Studienabschluss, wichtige Weiterbildungen und Zertifikate, Meisterbrief, Promotion, Approbation, Renteneintritt)
Relo	*Relocation* (also temporary, e.g., due to stays abroad)	*Umzug* (auch vorübergehend, z. B. durch Auslandsaufenthalte)
House	*Considerable change in domestic/family situation* (e.g., moving in with a partner, new family member in the household, housing expansion, house construction)	*Wesentliche Veränderung der häuslichen/familiären Situation* (z. B. Zusammenziehen mit Partner, neues Familienmitglied im Haushalt, Wohnraumausbau, Hausbau)
Confl	*Serious conflict with other people* (e.g., with family members, friends, classmates, colleagues, superiors, neighbors)	*Ernster Konflikt mit anderen Personen* (z. B. mit Familienangehörigen, Freunden, Mitschülern, Kollegen, Vorgesetzten, Nachbarn)
Rel‐	*Breaking up/loss of a close relationship/partnership* (e.g., due to separation, divorce, death of the partner)	*Lösen/Verlust einer ernsthaften Bindung/Partnerschaft* (z. B. durch Trennung, Scheidung, Tod des Partners)
Rel+	*Entering a close relationship/partnership*	*Eingehen einer ernsthaften Bindung/Partnerschaft*
Child	*Pregnancy/birth of own child/adoption of a child*	*Schwangerschaft/Geburt des eigenen Kindes/Adoption eines Kindes*
Soc+	*More social activities during leisure time* (e.g., spending more time with friends/partner/family, more volunteer or club activities)	*Mehr soziale Aktivitäten in der Freizeit* (z. B. mehr Zeit mit Freunden/Partner/der Familie verbringen, mehr ehrenamtliche oder Vereinstätigkeiten)
Soc‐	*Fewer social activities during leisure time* (e.g., spending less time with friends/partner/family, fewer volunteer or club activities)	*Weniger soziale Aktivitäten in der Freizeit* (z. B. weniger Zeit mit Freunden/Partner/der Familie verbringen, weniger ehrenamtliche oder Vereinstätigkeiten)
IllnO	*Severe physical or mental illness, injury, or death of a loved one* (e.g., partner, child, parent, friend, miscarriage/stillbirth)	*Schwerwiegende körperliche oder psychische Krankheit, Verletzung oder Tod einer nahestehenden Person* (z. B. Partner, Kind, Elternteil, Freund, Fehlgeburt/Totgeburt)
PhyS	*Severe own physical illness or injury* (e.g., due to accident)	*Schwerwiegende eigene körperliche Erkrankung oder Verletzung* (z. B. durch Unfall)
PsyS	*Own mental illness or addiction* (e.g., depression, anxiety, burn‐out; alcohol or (prescription) drug abuse or dependence)	*Eigene psychische Erkrankung oder Suchtprobleme* (z. B. Depression, Ängste, Burn‐out, Alkohol‐, Drogen‐ bzw. Tablettenmissbrauch oder ‐abhängigkeit)
Succ	*Significant personal success* (e.g., winning a competition, reaching/achieving a personally important goal/work)	*Bedeutsamer persönlicher Erfolg* (z. B. Sieg bei einem Wettkampf, Erreichen/Schaffen eines persönlich wichtigen Ziels/Werkes)
Fail	*Significant personal failure* (e.g., defeat in a competition, unfulfilled desire to have children/miscarriage, setbacks in achieving an important goal)	*Bedeutsamer persönlicher Misserfolg* (z. B. Niederlage bei einem Wettkampf, unerfüllter Kinderwunsch/Fehlgeburt, Rückschläge beim Erreichen eines wichtigen Ziels)
Crime	*Experiencing a crime* (e.g., theft, fraud) and/or physical and emotional violence by intentional actions of others (e.g., by assault, bullying, terrorist attack)	*Erleben eines Verbrechens* (z. B. Diebstahl, Betrug) und/oder körperlicher und seelischer Gewalt durch absichtliches Handeln anderer (z. B. durch Überfall, Mobbing, Terroranschlag)
Catas	*Experiencing a life‐threatening situation* not intentionally caused by you or others (e.g., natural disaster, accident, epidemic)	*Erleben einer lebensbedrohlichen Situation*, die nicht absichtlich durch Sie oder andere herbeigeführt wurde (z. B. Naturkatastrophe, Unfall, Epidemie)

**TABLE B2:** Frequencies and descriptive statistics of perceptions of critical life events occurring between time 1 and 2 and between time 2 and 3.

LE	T1–T2			T2–T3		
	RF	Valence	Contr.	RF	Valence	Contr.
		*M* (SD)	*M* (SD)		*M* (SD)	*M* (SD)
Occ+	0.52	1.94 (0.90)	0.29 (0.63)	0.50	1.89 (0.91)	0.37 (0.63)
Occ−	0.10	−1.11 (1.37)	−0.27 (0.68)	0.11	−1.28 (1.31)	−0.32 (0.71)
Fin+	0.37	1.89 (0.94)	0.33 (0.69)	0.37	1.91 (0.90)	0.29 (0.69)
Fin−	0.09	−1.16 (1.37)	−0.14 (0.71)	0.09	−1.00 (1.19)	0.00 (0.82)
NewE	0.32	1.98 (1.15)	0.55 (0.61)	0.34	1.84 (1.18)	0.49 (0.59)
Degr	0.24	2.24 (0.83)	0.68 (0.51)	0.23	2.13 (0.97)	0.68 (0.48)
Relo	0.27	1.76 (1.32)	0.60 (0.57)	0.25	1.91 (1.24)	0.63 (0.55)
House	0.31	1.45 (1.42)	0.36 (0.69)	0.32	1.53 (1.45)	0.31 (0.70)
Confl	0.38	−1.30 (1.52)	−0.29 (0.64)	0.37	−1.27 (1.56)	−0.27 (0.63)
Bin−	0.17	−0.42 (1.82)	−0.04 (0.79)	0.15	−0.78 (1.93)	−0.34 (0.74)
Bin+	0.17	2.49 (0.81)	0.41 (0.61)	0.16	2.47 (0.84)	0.28 (0.62)
Child	0.04	2.52 (1.27)	0.09 (0.73)	0.06	2.67 (0.79)	0.06 (0.76)
Soz+	0.39	2.17 (0.86)	0.64 (0.51)	0.28	2.16 (0.87)	0.65 (0.53)
Soz−	0.13	−0.83 (1.46)	0.12 (0.61)	0.33	−1.51 (1.15)	−0.40 (0.67)
IllnO	0.31	−1.51 (1.37)	−0.66 (0.60)	0.29	−1.63 (1.31)	−0.58 (0.66)
PhyS	0.14	−1.05 (1.54)	−0.52 (0.64)	0.14	−1.48 (1.36)	−0.43 (0.71)
PsyS	0.12	−1.55 (1.45)	−0.44 (0.57)	0.14	−1.61 (1.29)	−0.39 (0.54)
Succ	0.34	2.28 (0.72)	0.49 (0.55)	0.30	2.27 (0.82)	0.52 (0.54)
Fail	0.10	−1.97 (1.11)	−0.24 (0.65)	0.09	−1.55 (1.23)	−0.20 (0.64)
Crime	0.07	−1.81 (1.19)	−0.40 (0.59)	0.07	−1.93 (1.08)	−0.68 (0.57)
Catas	0.05	−0.86 (1.76)	−0.39 (0.74)	0.24	−1.43 (1.27)	−0.70 (0.56)

*Note:* For Abbreviations of LE see Appendix Table B1. Valence is measured on a scale from −3 (*very negative*) to 3 (*very positive*), controllability measured on a scale from −1 (*uncontrollable*) to 1 (*controllable*).

Abbreviations: Contr., controllability; LE, life events; RF, relative frequencies.

## Appendix C Unmoderated Base‐Models

**TABLE C1:** Coefficients and fit measures of unmoderated base models.

	Honesty‐humility	Emotionality	Extraversion	Agreeableness	Conscientiousness	Openness
Fit measures						
*χ* ^2^	10.04	16.07	20.19	11.60	23.44	11.57
df	8	8	8	8	8	8
*p*	< 0.001	0.041	0.010	0.170	0.003	0.171
CFI	0.998	0.996	0.993	0.998	0.991	0.998
TLI	0.997	0.992	0.987	0.995	0.983	0.996
RMSEA	0.020	0.040	0.049	0.027	0.056	0.027
SRMR	0.018	0.013	0.019	0.016	0.020	0.014
Intercepts						
$\alpha_{1S}$	3.737	3.145	3.582	3.194	3.683	3.538
$\alpha_{2S}$	3.745	3.115	3.521	3.209	3.645	3.514
$\alpha_{3S}$	3.753	3.124	3.513	3.214	3.659	3.513
$\alpha_{1I}$	3.760	3.134	3.529	3.153	3.770	3.342
$\alpha_{2I}$	3.818	3.146	3.446	3.147	3.763	3.363
$\alpha_{3I}$	3.807	3.171	3.452	3.162	3.789	3.364
Factor loadings						
$\lambda_{1S}$	1	1	1	1	1	1
$\lambda_{2S}$	1.054	1.061	1.034	0.966	1.039	1.045
$\lambda_{3S}$	0.952	1.044	0.968	0.914	1.013	1.016
$\lambda_{1I}$	1	1	1	1	1	1
$\lambda_{2I}$	1.162	1.091	1.077	1.086	1.190	0.956
$\lambda_{3I}$	1.139	1	0.942	1.071	1.032	0.953
$\mathrm{Cor}\left(\xi_{S},\xi_{I}\right)$	0.527	0.760	0.764	0.640	0.699	0.776

*Note:* $\alpha_{\mathrm{tS}}$: Intercepts of self‐reports (*t*: time‐point), $\alpha_{\mathrm{tI}}$: Intercepts of informant‐reports, $\lambda_{\mathrm{tS}}$: Factor loadings of self‐reports, $\lambda_{\mathrm{tI}}$: Factor loadings of informant‐reports, $\xi_{S}$: Latent trait variable of self‐reports, $\xi_{I}$: Latent trait variable of informant‐reports.

Abbreviations: CFI, Comparative Fit Index; RMSEA, Root Mean Square Error of Approximation; SRMR, Standardized Root Mean Squared Residual; TLI, Tucker‐Lewis Index.

## Appendix D Regression Equations for Moderated Parameters of MNLST Models

In the first series of models, additive trait‐change parameters $\Delta_{\mathrm{St}}$ and $\Delta_{\mathrm{It}}$, and multiplicative trait‐change parameters $\lambda_{\mathrm{St}}$ and $\lambda_{\mathrm{It}}$ at t>1 (with S denoting self‐reports and I denoting informant‐reports) were regressed on gender, age, the occurrence of the respective $LE_{t}$ at time t, the gender × $LE_{t}$ interaction and the age × $LE_{t}$ interaction. Initial values (at time 1) $\alpha_{S}$ and $\alpha_{I}$ were defined as a function of gender and age. Effects of covariates ($b_{k}$) on trait change parameters were set equal across time. For trait‐change parameters at time 3, we also included the lagged effects of $LE_{2}$ as well as the $LE_{2}\times LE_{3}$ interaction (i.e., the effect of the repeated occurence of the respective event). As the trait factor loadings in LST‐R theory are assumed to be positive, the corresponding parameters are exponentiated to ensure positive loadings for all values of the covariates (Oeltjen et al. 2023; Steyer et al. 2015).

(D1)
$$\alpha_{S}=b_{0}^{\alpha_{S}}+b_{1}^{\alpha_{S}}X_{\text{Gender}}+b_{2}^{\alpha_{S}}X_{\mathrm{Age}}$$

(D2)
$$\alpha_{I}=b_{0}^{\alpha_{I}}+b_{1}^{\alpha_{I}}X_{\text{Gender}}+b_{2}^{\alpha_{I}}X_{\mathrm{Age}}$$

(D3)
$$\Delta_{S2}=b_{0}^{\Delta_{S2}}+b_{1}^{\Delta_{S}}X_{\text{Gender}}+b_{2}^{\Delta_{S}}X_{\mathrm{Age}}+b_{3}^{\Delta_{S}}X_{LE_{2}}+b_{4}^{\Delta_{S}}X_{\text{Gender}\times LE_{2}}+b_{5}^{\Delta_{S}}X_{\mathrm{Age}\times LE_{2}}$$

(D4)
$$\Delta_{I2}=b_{0}^{\Delta_{I2}}+b_{1}^{\Delta_{I}}X_{\text{Gender}}+b_{2}^{\Delta_{I}}X_{\mathrm{Age}}+b_{3}^{\Delta_{I}}X_{LE_{2}}+b_{4}^{\Delta_{I}}X_{\text{Gender}\times LE_{2}}+b_{5}^{\Delta_{I}}X_{\mathrm{Age}\times LE_{2}}$$

(D5)
$$\begin{array}{c} \Delta_{S3}=b_{0}^{\Delta_{S3}}+b_{1}^{\Delta_{S}}X_{\text{Gender}}+b_{2}^{\Delta_{S}}X_{\mathrm{Age}}+b_{3}^{\Delta_{S}}X_{LE_{3}}+b_{4}^{\Delta_{S}}X_{\text{Gender}\times LE_{3}}\\ +b_{5}^{\Delta_{S}}X_{\mathrm{Age}\times LE_{3}}+b_{6}^{\Delta_{S}}X_{LE_{2}}+b_{7}^{\Delta_{S}}X_{LE_{2}\times LE_{3}} \end{array}$$

(D6)
$$\begin{array}{c} \Delta_{I3}=b_{0}^{\Delta_{I3}}+b_{1}^{\Delta_{I}}X_{\text{Gender}}+b_{2}^{\Delta_{I}}X_{\mathrm{Age}}+b_{3}^{\Delta_{I}}X_{LE_{3}}+b_{4}^{\Delta_{I}}X_{\text{Gender}\times LE_{3}}\\ +b_{5}^{\Delta_{I}}X_{\mathrm{Age}\times LE_{3}}+b_{6}^{\Delta_{I}}X_{LE_{2}}+b_{7}^{\Delta_{I}}X_{LE_{2}\times LE_{3}} \end{array}$$

(D7)
$$\lambda_{S2}=\exp\left(b_{0}^{\lambda_{S2}}+b_{1}^{\lambda_{S}}X_{\text{Gender}}+b_{2}^{\lambda_{S}}X_{\mathrm{Age}}+b_{3}^{\lambda_{S}}X_{LE_{2}}+b_{4}^{\lambda_{S}}X_{\text{Gender}\times LE_{2}}+b_{5}^{\lambda_{S}}X_{\mathrm{Age}\times LE_{2}}\right)$$

(D8)
$$\lambda_{I2}=\exp\left(b_{0}^{\lambda_{I2}}+b_{1}^{\lambda_{I}}X_{\text{Gender}}+b_{2}^{\lambda_{I}}X_{\mathrm{Age}}+b_{3}^{\lambda_{I}}X_{LE_{2}}+b_{4}^{\lambda_{I}}X_{\text{Gender}\times LE_{2}}+b_{5}^{\lambda_{I}}X_{\mathrm{Age}\times LE_{2}}\right)$$

(D9)
$$\begin{array}{c} \lambda_{S3}=\exp\left(b_{0}^{\lambda_{S3}}+b_{1}^{\lambda_{S}}X_{\text{Gender}}+b_{2}^{\lambda_{S}}X_{\mathrm{Age}}+b_{3}^{\lambda_{S}}X_{LE_{3}}+b_{4}^{\lambda_{S}}X_{\text{Gender}\times LE_{3}}\right.\\ \left.+b_{5}^{\lambda_{S}}X_{\mathrm{Age}\times LE_{3}}+b_{6}^{\lambda_{S}}X_{LE_{2}}+b_{7}^{\lambda_{S}}X_{LE_{2}\times LE_{3}}\right) \end{array}$$

(D10)
$$\begin{array}{c} \lambda_{I3}=\exp\left(b_{0}^{\lambda_{I3}}+b_{1}^{\lambda_{I}}X_{\text{Gender}}+b_{2}^{\lambda_{I}}X_{\mathrm{Age}}+b_{3}^{\lambda_{I}}X_{LE_{3}}+b_{4}^{\lambda_{I}}X_{\text{Gender}\times LE_{3}}\right.\\ \left.+b_{5}^{\lambda_{I}}X_{\mathrm{Age}\times LE_{3}}+b_{6}^{\lambda_{I}}X_{LE_{2}}+b_{7}^{\lambda_{I}}X_{LE_{2}\times LE_{3}}\right) \end{array}$$

In the second series of models, additive trait‐change parameters $\Delta_{\mathrm{St}}$ and $\Delta_{\mathrm{It}}$, and multiplicative trait‐change parameters $\lambda_{\mathrm{St}}$ and $\lambda_{\mathrm{It}}$ at time t>1 were regressed on the individual perception of LE in terms of valence, controllability, and the valence × controllability interaction at time t. As event characteristics were only assessed in case of the occurrence of an event, these models could only be run with a subsample of individuals who actually experienced the respective event (and only for the most frequently occurring LE).

(D11)
$$\Delta_{S2}=b_{0}^{\Delta_{S2}}+b_{1}^{\Delta_{S}}X_{\text{Valenc}e_{2}}+b_{2}^{\Delta_{S}}X_{\text{Contro}l_{2}}+b_{3}^{\Delta_{S}}X_{\text{Valenc}e_{2}\times \text{Contro}l_{2}}$$

(D12)
$$\Delta_{I2}=b_{0}^{\Delta_{I2}}+b_{1}^{\Delta_{I}}X_{\text{Valenc}e_{2}}+b_{2}^{\Delta_{I}}X_{\text{Contro}l_{2}}+b_{3}^{\Delta_{I}}X_{\text{Valenc}e_{2}\times \text{Contro}l_{2}}$$

(D13)
$$\Delta_{S3}=b_{0}^{\Delta_{S3}}+b_{1}^{\Delta_{S}}X_{\text{Valenc}e_{3}}+b_{2}^{\Delta_{S}}X_{\text{Contro}l_{3}}+b_{3}^{\Delta_{S}}X_{\text{Valenc}e_{3}\times \text{Contro}l_{3}}$$

(D14)
$$\Delta_{I3}=b_{0}^{\Delta_{I3}}+b_{1}^{\Delta_{I}}X_{\text{Valenc}e_{3}}+b_{2}^{\Delta_{I}}X_{\text{Contro}l_{3}}+b_{3}^{\Delta_{I}}X_{\text{Valenc}e_{3}\times \text{Contro}l_{3}}$$

(D15)
$$\lambda_{S2}=\exp\left(b_{0}^{\lambda_{S2}}+b_{1}^{\lambda_{S}}X_{\text{Valenc}e_{2}}+b_{2}^{\lambda_{S}}X_{\text{Contro}l_{2}}+b_{3}^{\lambda_{S}}X_{\text{Valenc}e_{2}\times \text{Contro}l_{2}}\right)$$

(D16)
$$\lambda_{I2}=\exp\left(b_{0}^{\lambda_{I2}}+b_{1}^{\lambda_{I}}X_{\text{Valenc}e_{2}}+b_{2}^{\lambda_{I}}X_{\text{Contro}l_{2}}+b_{3}^{\lambda_{I}}X_{\text{Valenc}e_{2}\times \text{Contro}l_{2}}\right)$$

(D17)
$$\lambda_{S3}=\exp\left(b_{0}^{\lambda_{S3}}+b_{1}^{\lambda_{S}}X_{\text{Valenc}e_{3}}+b_{2}^{\lambda_{S}}X_{\text{Contro}l_{3}}+b_{3}^{\lambda_{S}}X_{\text{Valenc}e_{3}\times \text{Contro}l_{3}}\right)$$

(D18)
$$\lambda_{I3}=\exp\left(b_{0}^{\lambda_{I3}}+b_{1}^{\lambda_{I}}X_{\text{Valenc}e_{3}}+b_{2}^{\lambda_{I}}X_{\text{Contro}l_{3}}+b_{3}^{\lambda_{I}}X_{\text{Valenc}e_{3}\times \text{Contro}l_{3}}\right)$$

## Appendix E

The following non‐informative priors were used for the specification of MNLST models. The prior specifications apply to all covariates *k* and time points *t* in both series of models.

$$\begin{array}{l} b_{0}^{\alpha_{S}}\sim N\left(0,0.001\right)\\ b_{0}^{\alpha_{I}}\sim N\left(0,0.001\right)\\ b_{k}^{\alpha_{S}}\sim N\left(0,0.001\right)\\ b_{k}^{\alpha_{I}}\sim N\left(0,0.001\right)\\ b_{0}^{\Delta_{\mathrm{St}}}\sim N\left(0,0.001\right)\;\text{for}\;\;t>1\\ b_{0}^{\Delta_{\mathrm{It}}}\sim N\left(0,0.001\right)\;\text{for}\;\;t>1\\ b_{k}^{\Delta_{S}}\sim N\left(0,0.001\right)\;\text{for}\;\;t>1\\ b_{k}^{\Delta_{I}}\sim N\left(0,0.001\right)\;\text{for}\;\;t>1\\ b_{0}^{\lambda_{\mathrm{St}}}\sim N\left(1,0.001\right)\;\text{for}\;\;t>1\\ b_{0}^{\lambda_{\mathrm{It}}}\sim N\left(1,0.001\right)\;\text{for}\;\;t>1\\ b_{k}^{\lambda_{S}}\sim N\left(0,0.001\right)\;\text{for}\;\;t>1\\ b_{k}^{\lambda_{I}}\sim N\left(0,0.001\right)\;\text{for}\;\;t>1\\ \epsilon_{\mathrm{it}}\sim \mathrm{IG}\left(0.001,0.001\right)\\ \left[\begin{array}{c} \xi_{S}\\ \xi_{I} \end{array}\right]=\mathrm{MVN}\left(\mu ,T\right)\\ \text{with}\;\;T=\left[\begin{array}{cc} \tau_{S}^{2} & \\ \tau_{\mathrm{SI}} & \tau_{I}^{2} \end{array}\right],\;\mu =\begin{array}{c} \mu_{S}\\ \mu_{I} \end{array},\text{and}\\ Τ\sim \mathrm{IW}\left(\Psi ,v\right) \end{array}$$

where Ψ is a unit matrix of size d=2 with v=d+1=3. *N* denotes the normal distribution (with mean and precision), *MVN* the multivariate normal distribution, *IG* the inverse gamma distribution, and *IW* the inverse Wishart distribution. $\epsilon_{\mathrm{it}}$ denote measurement error variables, $\xi_{S}$ and $\xi_{I}$ are the latent trait variables of self‐ (*S*) and informant‐reports (*I*). Parameter constraints (for identification purposes) were imposed by specifying highly informative priors for the respective parameters (expectations of the latent trait variables $\mu_{S}$ and $\mu_{I}$ and factor loadings of the first time point $\lambda_{S1}$ and $\lambda_{I1}$):

$$\begin{array}{l} \mu_{S}\sim N\left(0,1^{10}\right)\\ \mu_{I}\sim N\left(0,1^{10}\right)\\ \lambda_{S1}\sim N\left(1,1^{10}\right)\\ \lambda_{I1}\sim N\left(1,1^{10}\right) \end{array}$$
